# Fragmentstein—facilitating data reuse for cell-free DNA fragment analysis

**DOI:** 10.1093/bioinformatics/btae017

**Published:** 2024-01-15

**Authors:** Zsolt Balázs, Todor Gitchev, Ivna Ivanković, Michael Krauthammer

**Affiliations:** Department of Quantitative Biomedicine, University of Zurich, Zurich 8057, Switzerland; Biomedical Informatics, University Hospital of Zurich, Zurich 8091, Switzerland; Department of Quantitative Biomedicine, University of Zurich, Zurich 8057, Switzerland; Biomedical Informatics, University Hospital of Zurich, Zurich 8091, Switzerland; Department of Quantitative Biomedicine, University of Zurich, Zurich 8057, Switzerland; Biomedical Informatics, University Hospital of Zurich, Zurich 8091, Switzerland; Department of Quantitative Biomedicine, University of Zurich, Zurich 8057, Switzerland; Biomedical Informatics, University Hospital of Zurich, Zurich 8091, Switzerland

## Abstract

**Summary:**

Method development for the analysis of cell-free DNA (cfDNA) sequencing data is impeded by limited data sharing due to the strict control of sensitive genomic data. An existing solution for facilitating data sharing removes nucleotide-level information from raw cfDNA sequencing data, keeping alignment coordinates only. This simplified format can be publicly shared and would, theoretically, suffice for common functional analyses of cfDNA data. However, current bioinformatics software requires nucleotide-level information and cannot process the simplified format. We present Fragmentstein, a command-line tool for converting non-sensitive cfDNA-fragmentation data into alignment mapping (BAM) files. Fragmentstein complements fragment coordinates with sequence information from a reference genome to reconstruct BAM files. We demonstrate the utility of Fragmentstein by showing the feasibility of copy number variant (CNV), nucleosome occupancy, and fragment length analyses from non-sensitive fragmentation data.

**Availability and implementation:**

Implemented in bash, Fragmentstein is available at https://github.com/uzh-dqbm-cmi/fragmentstein, licensed under GNU GPLv3.

## Introduction

Cell-free DNA (cfDNA) sequencing is revolutionizing non-invasive approaches to prenatal testing, cancer detection, and transplant monitoring (Norwitz and Levy 2013, Oellerich *et al.* 2020, Cisneros-Villanueva *et al.* 2022). Clinically relevant features obtained from cfDNA include point mutations, mutational signatures (Sanmamed *et al.* 2015), copy number variation, fragment lengths (Mouliere *et al.* 2018), end motifs (Moldovan *et al.* 2021), fragmentation patterns (Cristiano *et al.*, 2019), and nucleosome footprints (Snyder *et al.* 2016, Sun *et al.* 2019, Peneder *et al.* 2021). However, sharing human cfDNA sequencing data is limited due to the highly sensitive nature of genome sequence information, hampering the development of bioinformatics software for data analysis. Some data are not shared at all, due to missing consent from the participants for sharing their genomic information with other researchers or certain countries limiting access to genetic data of their citizens (e.g. Denmark), and some of the genomic data are available upon request aided by restricted access repositories such as dbGAP (Mailman *et al.* 2007), the European Genome-Phenome Archive (Freeberg *et al.* 2022), or the Japanese Genotype-Phenotype Archive (Kodama *et al.* 2015); however, getting access to data through these repositories is slow and circumstantial. While sequence data are sensitive, much of the data of interest to cfDNA research can be separated from sequence information. Analyses that do not use point-mutation data can be performed using only cell-free DNA fragment coordinates, and such fragmentation data can be publicly shared. FinaleDB (Zheng *et al.* 2021) is a dedicated database providing open access to de-identified fragment coordinates in a tabulated file format; however, non-sensitive could potentially be shared through any open repository. Currently available cfDNA analysis software is written to only process alignment files, even if the analysis was possible without the sensitive sequence information. To fill this gap, we developed Fragmentstein, a command-line tool that converts fragmentation data into sequence alignment files that can be processed by most contemporary cfDNA analysis software.

## Usage

Fragmentstein is implemented as a bash script. It converts a tabulated file (BED, BEDPE, or TSV) containing fragment coordinates into a paired-end alignment file using the sequence of the specified reference genome. For a graphical overview, see Supplementary Fig. S1. Even though Fragmentstein was developed with the purpose to facilitate cfDNA sequence data reuse, it can also be used to create paired-end alignment files from any BED or similarly formatted tabular file.

## Application

**Figure 1.**
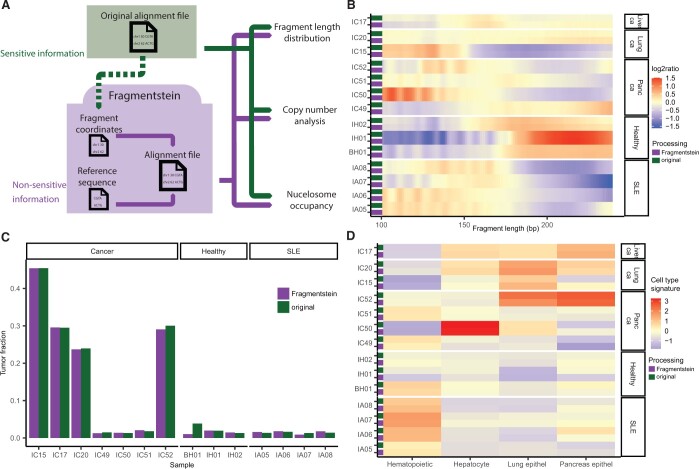
(A) Overview of the application test case. Alignment files and publicly accessible fragment coordinate data of the same samples were downloaded. Fragmentstein creates alignment files for each sample using only non-sensitive information. The original alignment files and the alignment files generated by Fragmentstein were subjected to fragment length, copy number, and nucleosome occupancy analysis. (B) Heatmap representation of fragment length distributions. The log2 ratio of fragments in each sample is depicted with red showing fragment sizes that are more and blue that are less frequent in a given sample. (C) Tumour fraction estimates output by ichorCNA based on copy number analysis. (D) Cell-type-specific nucleosome occupancy estimated by LIQUORICE. Signatures are defined as z-scores (compared to healthy samples) of coverage dip depths at cell-type-specific DHSs.

We evaluated the utility of Fragmentstein by analysing a commonly used cfDNA sequencing dataset (Snyder *et al.* 2016) in its original BAM format, containing nucleotide-level information, as well as in a non-sensitive TSV format from FinaleDB database (Zheng *et al.* 2021), containing sequence coordinates only. In order to demonstrate the feasibility of analyses on a range of different cfDNA data, we included paired-end sequencing data from both ssDNA and dsDNA libraries from the Snyder dataset. We analysed whole-genome cfDNA sequencing data of three healthy, four lupus erythematosus, and seven cancer samples sequenced to depths ranging from 10 to 60×. In order to evaluate the feasibility of different types of analyses with the outputs of our tool, we performed fragment length distribution analysis, copy number analysis (Adalsteinsson *et al.* 2017), and nucleosome profiling (Peneder *et al.* 2021) on both the original BAM files and the non-sensitive sequencing data processed by Fragmentstein (Fig. 1A). The bam files were processed using the same filtering settings (minimum mapping quality: 30); however, our pipeline for processing the “original” bam files obtained from the publication by Snyder *et al.* (2016) differed from the pipeline used by FinaleDB in three points: the FinaleDB pipeline used trimmomatic (Bolger *et al.* 2014) for read trimming and samblaster (Faust and Hall 2014) for marking duplicates, whereas our pipeline used skewer (Jiang *et al.* 2014) and picard (http://broadinstitute.github.io/picard/), respectively, and while the FinaleDB pipeline calculates GC bias, it does not correct for it, whereas our pipeline does (see the Supplementary Methods for more details).

Fragment length distributions were very similar when analysing the original BAM files and the BAM files output by Fragmentstein (Fig. 1B). It has been observed that in cancer and several inflammatory diseases, cfDNA fragments are shorter than in healthy individuals. Therefore, we compared the ratio of short (shorter than 150 bp) fragments in healthy individuals, lupus erythematosus, and cancer patients and received near identical results from original BAM files and the Fragmentstein outputs (Supplementary Fig. S2). The slight differences, most apparent in the ssDNA libraries, can be attributed to differences in the alignment filtering (see Supplementary Methods).

Using the ichorCNA package (Adalsteinsson *et al.* 2017), we detected the same copy number variants with similar tumour fraction estimates in both the original BAM files and the files processed with Fragmentstein (Fig. 1C and Supplementary Fig. S3).

We performed nucleosome footprint analysis as implemented in the LIQUORICE package (Peneder *et al.* 2021) to identify differences in cell-type signatures between samples. Cell-type signatures were defined as a drop in coverage at cell-type-specific DNase hypersensitivity sites (DHSs). We observed similar levels of contribution of the analysed cell types (haematopoietic, hepatocyte, lung epithelium, and pancreas epithelium) in the original BAM files and the ones created by Fragmentstein (Fig. 1D). While key observations such as increased haematopoietic signatures in SLE samples and decreased haematopoietic signatures in cancer samples were constant between the two processing methods, the intensity of the hepatocyte and pancreas epithelial signature was different. This observation highlights that some derived measures such as nucleosome footprints may be sensitive to preprocessing methods such as different alignment filtering or GC bias correction options.

## Discussion

Fragmentstein provides a simple and flexible solution to converting fragment coordinate information from non-sensitive cfDNA data to alignment (BAM) files which can be processed by typical bioinformatics software. While information such as mutational information and mapping quality data is lost during data de-identification when compared to the original BAM files, the recovered alignment files are suitable for most DNA fragment-based analyses. The script is openly accessible, easy to use, and can be customized to fit the user’s specific needs.

## Supplementary Material

btae017_Supplementary_Data

## Data Availability

Sequencing data used in the study have been acquired from FinalDB (http://finaledb.research.cchmc.org/; https://kircherlab.bihealth.org/download/cfDNA/) and from the Gene Expression Omnibus using the accession number GSE71378 (https://www.ncbi.nlm.nih.gov/geo/query/acc.cgi?acc=GSE71378).

## References

[btae017-B1] Adalsteinsson VA , Ha G, Freeman SS et al Scalable whole-exome sequencing of cell-free DNA reveals high concordance with metastatic tumors. Nat Commun 2017;8:1324. 10.1038/s41467-017-00965-y 29109393 PMC5673918

[btae017-B2] Bolger AM , Lohse M, Usadel B. Trimmomatic: a flexible trimmer for illumina sequence data. Bioinformatics 2014;30:2114–20. 10.1093/bioinformatics/btu170 24695404 PMC4103590

[btae017-B3] Cisneros-Villanueva M , Hidalgo-Pérez L, Rios-Romero M et al Cell-free DNA analysis in current cancer clinical trials: a review. Br J Cancer 2022;126:391–400. 10.1038/s41416-021-01696-0 35027672 PMC8810765

[btae017-B4] Cristiano S , Leal A, Phallen J et al Genome-wide cell-free DNA fragmentation in patients with cancer. Nature 2019;570:385–9. 10.1038/s41586-019-1272-6 31142840 PMC6774252

[btae017-B5] Faust GG , Hall IM. SAMBLASTER: fast duplicate marking and structural variant read extraction. Bioinformatics 2014;30:2503–5. 10.1093/bioinformatics/btu314 24812344 PMC4147885

[btae017-B6] Freeberg MA , Fromont LA, Teresa D’Altri AF et al The European genome-phenome archive in 2021. Nucleic Acids Res 2022;50:D980–D987. 10.1093/NAR/GKAB1059 34791407 PMC8728218

[btae017-B7] Jiang H , Lei R, Ding S-W et al Skewer: a fast and accurate adapter trimmer for next-generation sequencing paired-end reads. BMC Bioinform 2014;15:182. 10.1186/1471-2105-15-182 PMC4074385 24925680

[btae017-B8] Kodama Y , Mashima J, Kosuge T et al The DDBJ Japanese genotype-phenotype archive for genetic and phenotypic human data. Nucleic Acids Res 2015;43:D18–D22. 10.1093/NAR/GKU1120 25477381 PMC4383935

[btae017-B9] Mailman MD , Feolo M, Jin Y et al The NCBI dbGaP database of genotypes and phenotypes. Nat Genet 2007; 39:1181–6. 10.1038/ng1007 17898773 PMC2031016

[btae017-B10] Moldovan N , van der Pol Y, van den Ende T et al Genome-wide cell-free DNA termini in patients with cancer. medRxiv, 10.1101/2021.09.30.21264176, preprint: not peer reviewed.

[btae017-B11] Mouliere F , Chandrananda D, Piskorz AM et al Enhanced detection of circulating tumor DNA by fragment size analysis. Sci Transl Med 2018;10. 10.1126/scitranslmed.aat4921 PMC6483061 30404863

[btae017-B12] Norwitz ER , Levy B. Noninvasive prenatal testing: the future is now. Rev Obstet Gynecol 2013;6:48. 10.3909/riog0201 24466384 PMC3893900

[btae017-B13] Oellerich M , Christenson RH, Beck J et al Donor-derived cell-free DNA testing in solid organ transplantation: a value proposition. J Appl Lab Med 2020;5:993–1004. 10.1093/JALM/JFAA062 32447378

[btae017-B14] Peneder P , Stütz AM, Surdez D et al Multimodal analysis of cell-free DNA whole-genome sequencing for pediatric cancers with low mutational burden. Nat Commun 2021;12:3230–16. 10.1038/s41467-021-23445-w 34050156 PMC8163828

[btae017-B15] Sanmamed MF , Fernández-Landázuri S, Rodríguez C et al Quantitative cell-free circulating BRAFV600E mutation analysis by use of droplet digital PCR in the follow-up of patients with melanoma being treated with BRAF inhibitors. Clin Chem 2015;61:297–304. 10.1373/clinchem.2014.230235 25411185

[btae017-B16] Snyder MW , Kircher M, Hill AJ et al Cell-free DNA comprises an in vivo nucleosome footprint that informs its Tissues-Of-Origin. Cell 2016;164:57–68. 10.1016/j.cell.2015.11.050 26771485 PMC4715266

[btae017-B17] Sun K , Jiang P, Cheng SH et al Orientation-aware plasma cell-free DNA fragmentation analysis in open chromatin regions informs tissue of origin. Genome Res 2019;29:418–27. 10.1101/GR.242719.118 30808726 PMC6396422

[btae017-B18] Zheng H , Zhu MS, Liu Y. FinaleDB: a browser and database of cell-free DNA fragmentation patterns. Bioinformatics 2021;37:2502–3. 10.1093/BIOINFORMATICS/BTAA999 33258919 PMC8388032

