# Supplementary material for: Fragmentstein—facilitating data reuse for cell-free DNA fragment analysis
Source: Bioinformatics. 2024 Jan 15;40(1):btae017. doi: 10.1093/bioinformatics/btae017 (PMC10805340; doi:10.1093/bioinformatics/btae017)
Supplement: btae017_Supplementary_Data [file btae017_supplementary_data.pdf]

Fragmentstein - Facilitating data reuse for cell-free DNA fragment analysis

Zsolt Balázs*, Todor Gitchev, Ivna Ivankovic, Michael Krauthammer*

Department of Quantitative Biomedicine, University of Zurich, 8057 Zurich, Switzerland

Biomedical Informatics, University Hospital of Zurich, 8057 Zurich, Switzerland

*Corresponding author. Biomedical Informatics, University Hospital of Zurich, Schmelzbergstrasse 26, 8057 Zurich, Switzerland. E-mail: zsolt.balazs@uzh.ch, michael.krauthammer@uzh.ch

Supplementary Material

Supplementary Methods

Data acquisition

14 samples from the publication by Snyder et al. (Snyder *et al.*, 2016) were downloaded from the website, https://kircherlab.bihealth.org/download/cfDNA/. Note, that data of the same samples can also be accessed through the Gene Expression Omnibus (GEO) using the accession number GSE71378 (https://www.ncbi.nlm.nih.gov/geo/query/acc.cgi?acc=GSE71378), however, the bam files downloaded there have a slightly different read composition. The corresponding samples' fragment .tsv files (hg38 genome build) were downloaded from FinaleDB (Zheng *et al.*, 2021) (http://finaledb.research.cchmc.org/). We chose the 14 samples from the dataset that contained data of more than 100 million fragments per sample.

Processing sensitive data files

Reads were extracted from the bam files using the following samtools (Danecek *et al.*, 2021) command: `samtools sort -n $BAM -o ${BAM}.sorted.bam`, `samtools fastq -o ${BAM}.sorted.bam -1 ${BAM}_R1.fastq.gz -2 ${BAM}_R2.fastq.gz`, trimmed using skewer (`skewer -Q 30 -q 35 -l 50 `) and subsequently mapped to the human genome (GCA_000001405.15_GRCh38 build) using bwa-mem. Alignments with mapping quality lower than 30 were discarded. Unmapped reads and secondary alignments were also discarded using samtools. Duplicates were removed using picard (http://broadinstitute.github.io/picard/). GC bias correction was performed using the correctGCBias function of deeptools (v3.5.0) (Ramírez *et al.*, 2016). These BAM files are referred to as "original" BAM files in the text.

Processing publicly available data with Fragmentstein

The fragment.tsv files downloaded from FinaleDB were filtered for fragments with a quality score of 30 (`samtools view -h 256 -q 30 -x XA `) and above and converted to BAM files using Fragmentstein and the GCA_000001405.15_GRCh38_no_alt_analysis_set genome build. Base quality scores were set to Q37 (PHRED score for the letter "F").

Fragment length analysis

Length features were extracted both from the original BAM files and the ones created by Fragmentstein using the following command:
`samtools view $sample | grep -E "chr[1-9]" | cut -f 9 | sort | uniq -c | awk '$2>0{print $2,$1}'> /cluster/work/medinfmk/cfDNA-Snyder/resu/orig/${sample/.sortByCoord.bam/_hist.csv}` The distribution of fragments of length 101-225 bp was plotted in Figure 1B.

Copy number analysis

Copy number analysis was performed by ichorCNA (Adalsteinsson *et al.*, 2017) using the healthy (BH01, IH01, IH02) and SLE (IA05, IA06, IA07, IA08) samples as copy number neutral controls. To get the read's counts, we used the following readCounter command from the HMM Copy Utils (https://github.com/shahcompbio/hmmcopy_utils): `readCounter -w 1000000 -q 20 -c chr1,chr2,chr3,chr4,chr5,chr6,chr7,chr8,chr9,chr10,chr11,chr12,chr1 3,chr14,chr15,chr16,chr17,chr18,chr19,chr20,chr21,chr22,chrX,chrY`. The command used for running ichorCNA was: `runIchorCNA.R -normalPanel {input.pon} "c(0.95, 0.99, 0.995, 0.999)" "c(2)" "c(1:22)" "c(1:22)" 3 --genomeBuild hg38`.

Nucleosome footprint analysis

The LIQUORICE (Peneder *et al.*, 2021) algorithm was used to perform nucleosome footprint analysis on the BAM files. Cell-type signatures were defined as drops in coverage at cell-type specific DNase hypersensitivity sites (DHSs). Hematopoietic cell-specific DHS region sets were downloaded from the Supplementary dataset 8 of Peneder and his colleagues' publication. Hepatocyte, lung and pancreas epithelium-specific DHS regions were acquired from the Regulatory Elements Database (http://dnase.genome.duke.edu/) and are available together with the source code used for the analyses at https://github.com/uzh-dqbm-cmi/fragmentstein-paper.

References

Adalsteinsson,V.A. *et al.* (2017) Scalable whole-exome sequencing of cell-free DNA reveals high concordance with metastatic tumors. *Nat. Commun. 2017 81*, **8**, 1–13.

Danecek,P. *et al.* (2021) Twelve years of SAMtools and BCFtools. *Gigascience*, **10**, 1–4.

Peneder,P. *et al.* (2021) Multimodal analysis of cell-free DNA whole-genome sequencing for pediatric cancers with low mutational burden. *Nat. Commun. 2021 121*, **12**, 1–16.

Ramírez,F. *et al.* (2016) deepTools2: a next generation web server for deep-sequencing data analysis. *Nucleic Acids Res.*, **44**, W160–W165.

Snyder,M.W. *et al.* (2016) Cell-free DNA Comprises an In Vivo Nucleosome Footprint that Informs Its Tissues-Of-Origin. *Cell*, **164**, 57–68.

Zheng,H. *et al.* (2021) FinaleDB: a browser and database of cell-free DNA fragmentation patterns. *Bioinformatics*, **37**, 2502–2503.

Supplementary Figures

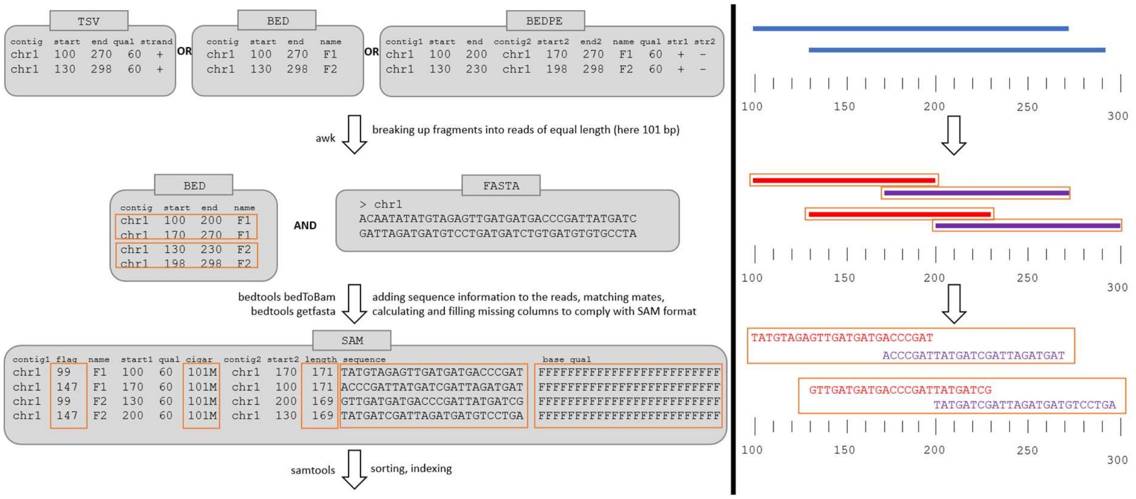

Supplementary Figure 1. Overview of the implementation of the algorithm. The left side shows an example of the file formats whereas the right side of the figure shows a graphical representation of the files on the left. Fragmentstein requires a fragment coordinate file in BED, BEDPE or in TSV format (following the FinaleDB specifications) and a reference FASTA file. Compressed and uncompressed input files are equally accepted. Fragmentstein breaks up fragments to create equal-size read pairs, where the size of the read is specified by the user. Paired read coordinates are converted to alignment file format using bedtools bedToBam function. The corresponding genome sequences are queried using bedtools getfasta function and inserted into the BAM file. The user can opt to remove special nucleotide notations that fall outside the commonly used set of characters in the FASTA file (A, C, G, T and N). This may be necessary, as some algorithms (such as LIQUORICE) do not accept BAM files containing incompletely specified nucleotides. The corresponding reads are paired, and the template length is calculated by the algorithm. Base-quality scores are set by the user. Finally, a header is added, and the alignment file is saved as a BAM file (not shown). The user can opt to sort and index the output BAM file.

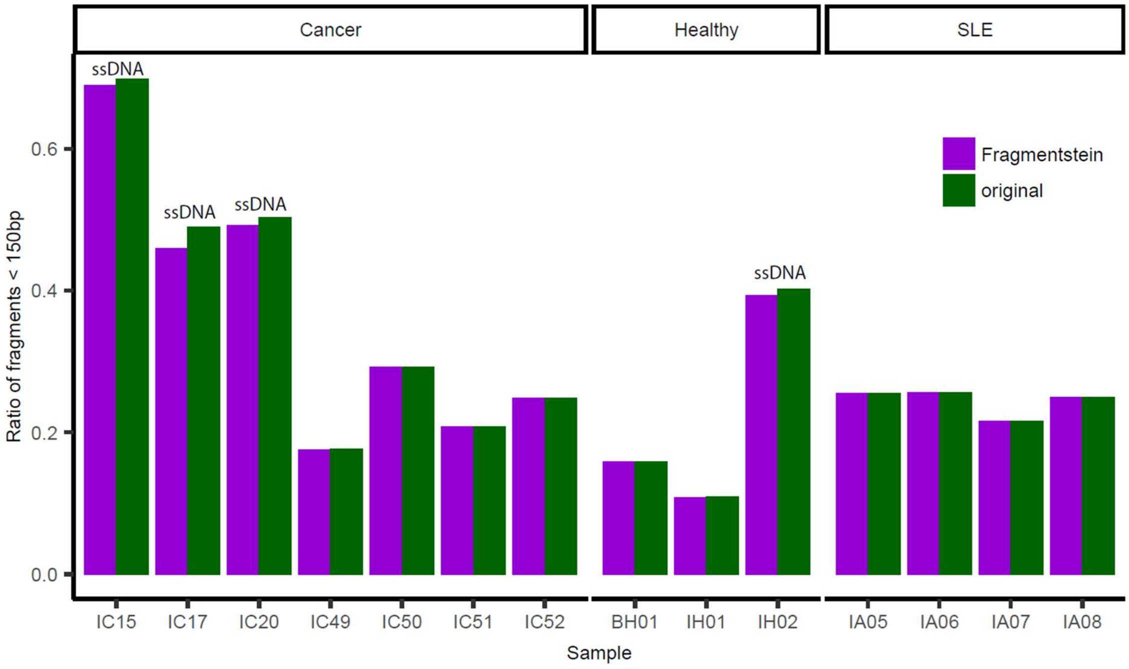

Supplementary Figure 2. Ratio of fragments that are shorter than 150 bp. Single-stranded DNA libraries are marked as ssDNA.

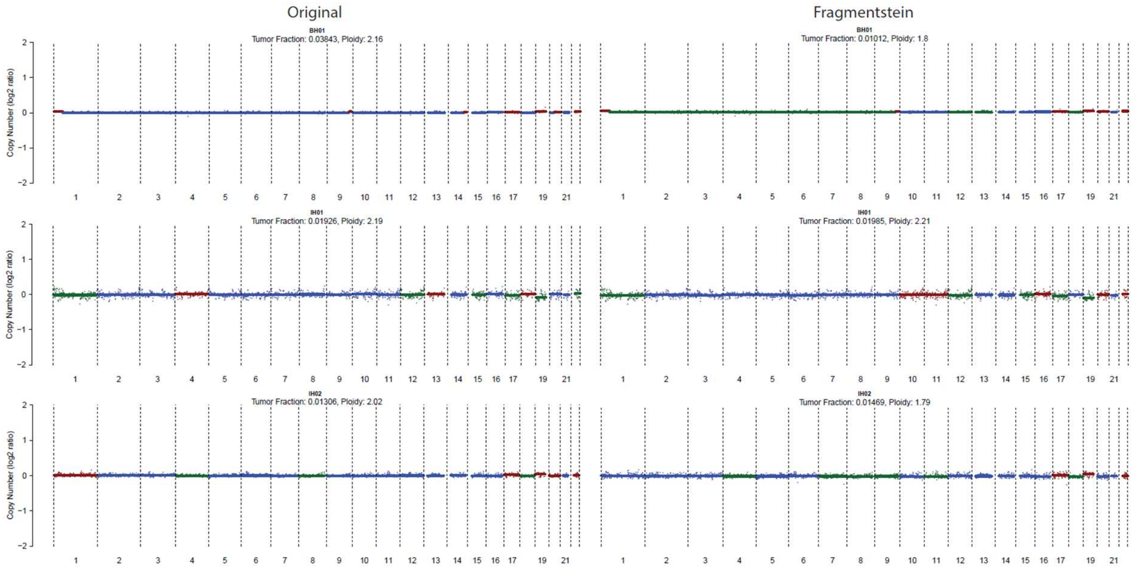

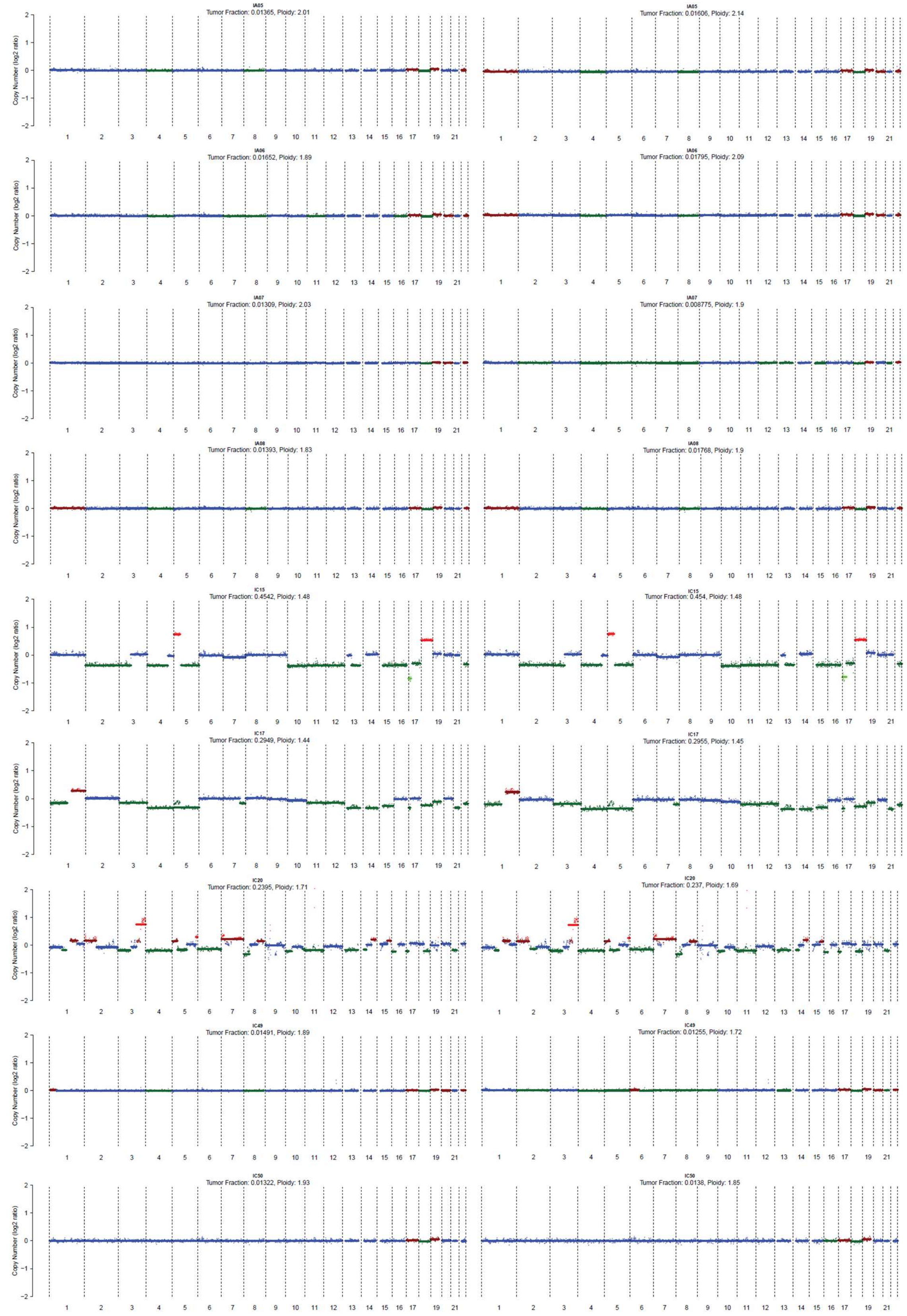

IA05
Tumor Fraction: 0.01365, Ploidy: 2.01
IA05
Tumor Fraction: 0.01606, Ploidy: 2.14
IA06
Tumor Fraction: 0.01652, Ploidy: 1.89
IA06
Tumor Fraction: 0.01795, Ploidy: 2.09
IA07
Tumor Fraction: 0.01309, Ploidy: 2.03
IA07
Tumor Fraction: 0.008775, Ploidy: 1.9
IA08
Tumor Fraction: 0.01393, Ploidy: 1.83
IA08
Tumor Fraction: 0.01768, Ploidy: 1.9
IC15
Tumor Fraction: 0.4542, Ploidy: 1.48
IC15
Tumor Fraction: 0.454, Ploidy: 1.48
IC17
Tumor Fraction: 0.2949, Ploidy: 1.44
IC17
Tumor Fraction: 0.2955, Ploidy: 1.45
IC20
Tumor Fraction: 0.2395, Ploidy: 1.71
IC20
Tumor Fraction: 0.237, Ploidy: 1.69
IC49
Tumor Fraction: 0.01491, Ploidy: 1.89
IC49
Tumor Fraction: 0.01255, Ploidy: 1.72
IC50
Tumor Fraction: 0.01322, Ploidy: 1.93
IC50
Tumor Fraction: 0.0138, Ploidy: 1.85
Copy Number (log2 ratio)

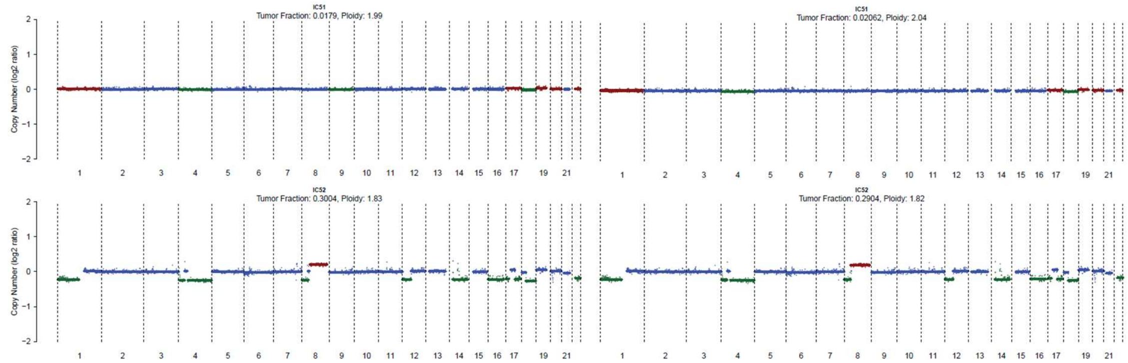

Supplementary Figure 3. Copy number analysis of healthy individuals (BH01, IH01, IH02), SLE (IA05, IA06, IA07, IA08) and cancer patients (IC15, IC17, IC20, IC49, IC50, IC51, IC52) using the original BAM files (left) and the BAM files generated by Fragmentstein (right).
